# An allostatic mechanism for M2 pyruvate kinase as an amino-acid sensor

**DOI:** 10.1042/BCJ20180171

**Published:** 2018-05-31

**Authors:** Meng Yuan, Iain W. McNae, Yiyuan Chen, Elizabeth A. Blackburn, Martin A. Wear, Paul A.M. Michels, Linda A. Fothergill-Gilmore, Ted Hupp, Malcolm D. Walkinshaw

**Affiliations:** 1 Centre for Translational and Chemical Biology, School of Biological Sciences, University of Edinburgh, Michael Swann Building, Max Born Crescent, Edinburgh EH9 3BF, U.K.; 2 Cell Signalling Unit, p53 Signal Transduction Laboratories, Institute of Genetics and Molecular Medicine, University of Edinburgh Cancer Research Centre, Edinburgh EH4 2XR, U.K.

**Keywords:** allostatic regulation, amino-acid regulation, enzyme mechanism, pyruvate kinase

## Abstract

We have tested the effect of all 20 proteinogenic amino acids on the activity of the M2 isoenzyme of pyruvate kinase (M2PYK) and show that, within physiologically relevant concentrations, phenylalanine, alanine, tryptophan, methionine, valine, and proline act as inhibitors, while histidine and serine act as activators. Size exclusion chromatography has been used to show that all amino acids, whether activators or inhibitors, stabilise the tetrameric form of M2PYK. In the absence of amino-acid ligands an apparent tetramer–monomer dissociation *K*_d_ is estimated to be ∼0.9 µM with a slow dissociation rate (*t*_1/2 _∼_ _15 min). X-ray structures of M2PYK complexes with alanine, phenylalanine, and tryptophan show the M2PYK locked in an inactive T-state conformation, while activators lock the M2PYK tetramer in the active R-state conformation. Amino-acid binding in the allosteric pocket triggers rigid body rotations (11°) stabilising either T or R states. The opposing inhibitory and activating effects of the non-essential amino acids serine and alanine suggest that M2PYK could act as a rapid-response nutrient sensor to rebalance cellular metabolism. This competition at a single allosteric site between activators and inhibitors provides a novel regulatory mechanism by which M2PYK activity is finely tuned by the relative (but not absolute) concentrations of activator and inhibitor amino acids. Such ‘allostatic’ regulation may be important in metabolic reprogramming and influencing cell fate.

## Introduction

In the 1920s, Otto Warburg observed that cancer cells produce ATP mainly through the glycolytic pathway, rather than oxidative phosphorylation, even under aerobic conditions [1]. This metabolic reprogramming favours proliferation of tumour cells by supporting metabolite synthesis and the accumulation of biomass [2,3]. The M2 isoenzyme of pyruvate kinase (M2PYK) has been shown to play important roles in metabolic reprogramming during cancer cell proliferation [4–6] and is a potential cancer therapeutic target [7,8]. The altered metabolic requirements of cancer cells are necessitated by periods of both quiescence and rapid growth. In the present paper, we introduce the idea that metabolic enzymes can act as ‘allostatic’ regulators with the ability to sense and react to fluctuations in the nutrient environment. We use the biological definition of allostasis to describe responses or mechanisms whereby the cell (or organism) accommodates changes (e.g. stress, nutrition) in readiness for adopting or moving to a different state (e.g. cell division) [9]. These changes are subtly different from homeostatic responses that react to changes in cell state to restrain or return the cell to its standard state (e.g. using feedback mechanisms) [9].

**Figure 1.**
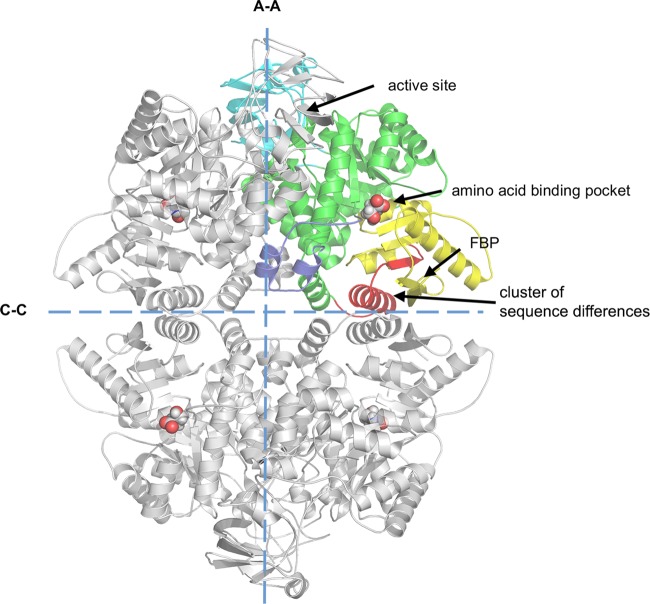
Crystal structure of M2PYK. M2PYK is a homotetramer that consists of an A-domain (green, residues 44–116 and 219–402); B-domain (cyan, residues 117–218); C-domain (yellow, residues 403–531); and N-terminal domain (blue, residues 13–43. Residues 1–12, as well as additional residues from the His_6_-tagged construct that were too disordered to be solved and shown in the structures). The only unconserved region between M1PYK and M2PYK is highlighted in red. Positions of the active site, the amino-acid binding pocket, and the FBP-binding pocket are also highlighted. The large (A–A) and small (C–C) interfaces are shown as dashed lines.

Pyruvate kinases (PYKs) catalyse the last step of the glycolytic pathway by transferring a phospho group from phosphoenolpyruvate (PEP) to ADP, thereby generating pyruvate and ATP. There are four PYK isoforms found in mammals: alternative RNA splicing allows the *PKLR* gene to encode LPYK in the liver and RPYK in red blood cells [10], while alternative RNA splicing of the *PKM* gene gives the M2PYK isoform, which is found in growing and dividing cells and the M1PYK (M1 isoenzyme of pyruvate kinase) isoform which is found in muscle tissues. M1PYK and M2PYK differ by only 22 amino acids (on the C–C interfaces, shown in Figure 1, highlighted in red) [11]. Each subunit of this homotetramer (Figure 1) consists of four domains: A, B, C, and N-terminal. The A-domain and C-domain correspond to the rigid body core of PYK. The active site is formed by a pocket between the rigid body core and the flexible B-domain.

M1PYK is the constitutively active form of the enzyme and despite the high (96%) overall sequence identity, M2PYK has a low basal activity but is activated by the allosteric effector fructose 1,6-bisphosphate (FBP) [12]. Structural studies of M1 and M2PYK show that the 22 different residues on the C–C interface form part of the tight inter-subunit interface, and facilitate the dissociation of M2PYK from an active tetramer to inactive monomers [13]. Monomers of M2PYK have been shown to translocate into the cell nucleus [14] and potentially act as protein kinases to affect transcription [15]. Regulatory post-translational modifications have been shown for M2PYK but not for M1PYK. For example, phosphorylation of residue Tyr105 inhibits tetramer formation of M2PYK [16], and Cys358 oxidation inhibits M2PYK activity and promotes the metabolic changes required for proliferation [17]. Acetylation of Lys305 inhibits M2PYK activity [18], and acetylation of Lys433 affects FBP binding and thus prevents M2PYK activation [19]. Hydroxylation of Pro403 and Pro408 promotes HIF-1 transactivation in cancer cells [20].

A chemically diverse collection of natural products and synthetic drug-like compounds have been shown to modulate M2PYK activity [13,21–27] and stimulate [13] or inhibit cell growth [23,28]. Regulatory effects of amino acids on the constitutively fully active M1PYK have been extensively studied [29–37]. The results showed that only phenylalanine moderately regulates M1PYK by a non-competitive inhibition of its enzymatic activity [29,33]. All the other amino acids have little direct regulatory effects on M1PYK. In contrast, M2PYK is known to respond to a wider range of effector molecules such as the inhibitory effect of phenylalanine [13] and activating effect of serine [24]. An interesting link has been shown between the level of expression of M2PYK and over 30 proteins which regulate amino-acid synthesis and metabolism [38]. Low M2PYK levels were shown to correlate with high levels of glutamic acid and glutamine but with low levels of seven other amino acids including histidine and serine, leading to the suggestion that PYK is a dosage-dependent regulator of cellular amino-acid homeostasis [39].

Here, we systematically investigated the effects of all 20 proteinogenic amino acids on M1PYK and M2PYK. A series of co-crystal complexes show that the amino acids bind in an allosteric pocket (found in both M1PYK and M2PYK; Figure 1). Differences in side chain interactions explain how all amino acids stabilise the tetrameric form of M2PYK and further show how they can stabilise either the inactive T-state (phenylalanine, alanine, tryptophan) or the active R-state (serine, histidine). Analytical gel chromatography and ELISA (enzyme-linked immunosorbent assay) were used to investigate the time-dependent dissociation of M2PYK and the stabilising effect of amino acids on the tetrameric form. These biochemical and biophysical stability studies along with the X-ray structures provide a molecular mechanism for allosteric regulation of M2PYK which is used by the cell to measure the level of nutrient. The fast (T to R allosteric switch) or slow (tetramer–monomer dissociation) responses of M2PYK provide molecular mechanisms which can be used by the cell to balance the rate of catabolism against nucleic acid and phospholipid anabolism as required for (cancer) cell proliferation [5,6]. Proliferating tumour cells undergo large fluctuations in nutrients depending on the stage of cell cycle and the local environment. We show here how M2PYK can act as an ‘allostatic’ regulator’ allowing the cell to adapt to changes in metabolite concentrations during stages in cell growth and cell division that require supplies of amino acids for protein and DNA production and at the same time require a regulated supply of pyruvate for ATP production. The unique competitive allosteric inhibitory and activating effects of the amino acids on M2PYK enzyme activity depend on their relative (rather than absolute) concentrations. Thus, the effect of amino-acid inhibitors (e.g. Ala and Phe) even at relatively high concentrations can be trumped by high concentrations of activators and *vice versa*. In contrast, relatively low concentrations of just one activator (or inhibitor) in the absence of a competing signal will control enzyme activity. The enzymatic response of M2PYK to these multiple complex signals provides the cell with an allostatic mechanism to facilitate changes required to adapt to either rapidly dividing or senescent states.

## Experimental procedures

### Expression and purification of human PYKs

Protein expression and purification were performed as described previously [13] with minor modifications. In brief, cDNA encoding human M1PYK and M2PYK with N-terminal His tags were codon-optimised for *Escherichia coli* expression and inserted into pET28a vectors. Afterwards, competent *E. coli* cells BL21(DE3) (from Novagen, Madison, WI) were transformed with these recombinant plasmids. Cells were grown in 2× tryptone yeast extract (tryptone and yeast extract were from Formedium, Hunstanton, U.K.) until an OD_600_ of 0.8–1.0, followed by a cold shock at 4°C for 1 h. The expression was induced at 18°C with 1 mM isopropyl-β-d-thiogalactopyranoside (purchased from Melford, Ipswich, U.K.). All media were provided with 50 µg/ml kanamycin (Melford). The cells were harvested, followed by lysing with a constant cell disruption system in lysis buffer [50 mM NaH_2_PO_4_, 300 mM NaCl, 20 mM imidazole (pH 8.0), and one one EDTA-free protease inhibitor tablet per 5 g of cell mass (from Roche, Welwyn Garden City, U.K.)] at 25 MPa. The lysate was centrifuged at 10°C for 45 min, and the supernatant was filtered through a 0.2-µm syringe filter. The supernatant was purified with an immobilised metal affinity HiTrap HP Sepharose column (precharged with cobalt), followed by a size-exclusion step with a Superdex 200 10/300GL column (all chromatography columns used in the present study were purchased from GE Healthcare Life Sciences, Buckinghamshire, U.K.) in Dulbecco's PBS without calcium and magnesium [Dulbecco's phosphate-buffered saline without calcium and magnesium (PBS-CM), from Sigma–Aldrich, Dorset, U.K.]. His_6_M1/2PYKs were concentrated to 20 mg/ml using a Vivaspin column (molecular mass cut-off, 30 kDa, from Sartorius, Goettingen, Germany) and stored at −80°C after flash-freezing in liquid nitrogen.

### Measurement of PYK activity

For the identification of PYK activity modulators, enzymatic activity assays were performed as follows. A 1.9 µl aliquot of 20 mg/ml M1 or M2PYK in PBS-CM was added individually into 5 ml of different amino acids at 5 mM in PBS-CM at pH 7.4 followed by an incubation at 37°C for 10 min. Then, the same volume of assay buffer [PBS-CM supplemented with 10 mM MgCl_2_, 100 mM KCl, 1 mM NADH, 40 µmol/min ml lactate dehydrogenase (LDH), as well as sub-saturating substrates PEP (1 mM) and ADP (0.8 mM)] was added into the protein solution and mixed (so the concentrations of all reagents were halved). The decrease in absorbance at 340 nm (the absorbance of NADH) was measured for 5 min at 37°C using a plate reader. PEP, ADP, LDH, NADH, FBP and all amino acids were from Sigma–Aldrich.

For the determination of kinetics of M2PYK in the absence/presence of amino acid modulators, enzymatic activity assays were performed as follows. A 5 µl aliquot of 0.002 mg/ml M2PYK in PBS-CM was incubated with different concentrations of amino acids in PBS-CM supplemented with 1 mM dithiothreitol at 37°C at pH 7.4 for 10 min. Then, the same volume of assay buffer [PBS-CM supplemented with 10 mM MgCl_2_, 100 mM KCl, 1 mM NADH, 40 µmol/min ml LDH, as well as saturating ADP (4 mM) and titrated substrate PEP (0.15–10 mM)] was added. Binding affinities of amino acid ligands (alanine, phenylalanine, tryptophan, and serine) against M2PYK were determined in the presence of a sub-saturating concentration of PEP (0.5 mM) and ADP (0.4 mM). For these uncompetitive inhibitors the *K_i_* values were calculated to be equal to IC_50_/2.

To investigate the competition between serine (activator) and alanine/phenylalanine (inhibitors), M2PYK was incubated with mixtures of serine and alanine (or serine and phenylalanine) over a range of different concentrations under the same condition described above. The decrease in absorbance at 340 nm was measured for 5 min at 37°C using a plate reader.

Values of the kinetic parameters (*V*_max_, *K*_0.5_(PEP), and *n*_H_) were determined by fitting the equation below to the data.

$$V=\frac{V_{\max}\times {[\mathrm{PEP}]}^{n_{H}}}{K_{0.5}^{n_{H}}+{\left[\mathrm{PEP}\right]}^{n_{H}}}$$

The Lineweaver–Burk plots were fit using the equation below:

$$Y=\frac{K{{}_{0.5}^{n_{H}}}_{}^{}+{\left(1/x\right)}^{n_{H}}}{V_{\max}\times {\left(1/x\right)}^{n_{H}}}$$

The Berkley Madonna software (http://www.berkeleymadonna.com/) was used to model approximate proportions of monomer and tetramer M2PYK at given total protein concentrations. The calculation was based on a simplified tetramer [T] monomer [M] equilibrium:

$$\left[T\right]⇌\left[M]+[M]+[M]+[M\right]\;\mathrm{with}\;K_{d}={\left[M\right]}^{4}/[T]$$

Monitoring the rate of loss of enzyme activity gave an estimated half-life (*t*_1/2_) of ∼15 min and a dissociation rate constant (*k*_off _= 0.69/*t*_1/2_) of ∼10^−3^ s^−1^. The association rate constant was scaled to give a *K*_d_ of 0.9 µM, which was estimated from analytical gel chromatography results.

### Analytical gel chromatography

Analytical gel chromatography was performed to characterise the oligomerisation/dissociation of M2PYK in the presence/absence of different amino acids. Aliquots of 400 µl of 0.1 mg/ml purified M2PYK were incubated with or without 10 mM amino acids in PBS-CM at room temperature at pH 7.4 overnight. After the incubation, samples were loaded independently onto a Superdex 200 PC 3.2/30 gel-filtration column. Samples of 25 µl were injected, and the column flow rate was maintained at 0.1 ml/min. Protein peaks were monitored using the absorbance at both 280 and 214 nm. The retention volumes for the M2PYK peaks were the same as those identified by SEC-MALS as monomer and tetramer peaks (using the same Superdex 200 column) in our previous study [13].

### Enzyme-linked immunosorbent assay

For the production of specific antibodies, a peptide immunogen (Supplementary Figure S1) specific for M2PYK was covalently conjugated with the carrier protein BSA, and injected into Balb/c female mice (6–7 weeks) at 2-week intervals. A mouse showing best selectivity for M2PYK was chosen as a spleen donor. After the production of a hybridoma, a cell line secreting a specific monoclonal antibody was screened and expanded. The specific monoclonal antibody against M2PYK was identified, purified and conjugated with horseradish peroxidase (HRP) for further ELISAs.

A stock solution of anti-His-tag antibody in carbonate buffer (pH 9.6) was coated onto wells at 50 µl/well at 4°C overnight. The wells were then blocked with 100 µl/well of PBS/Tween 20/3% BSA for 1 h at room temperature. Titrated M2PYK (2.5–0.0039 µg/ml) was incubated with or without ligands (10 mM for all amino acids and 1 mM for FBP) in PBS/Tween 20/3% BSA at room temperature for 1 h. Then, 100 µl of the solution was added to each prepared well for an incubation of 1 h. Afterwards, a 100 µl aliquot of PBS/Tween 20/3% BSA containing 4 µg/ml HRP-conjugated M2PYK antibody was added to each well and incubated for 1 h at room temperature. Electrochemiluminescence solution was added at 50 µl/well to develop the luminescence, which was read immediately with a plate reader. Three washes with PBS/Tween 20/3% BSA were performed between each step.

### Crystallisation and data collection

Single crystals of M2PYK were obtained at 17°C by vapour diffusion using the hanging-drop technique. The drops were formed by mixing 1.5 µl of wild-type M2PYK (20 mg/ml) solution containing ligands with 1.5 µl of reservoir solution (composed of 11–16% PEG 3350, 100 mM sodium cacodylate, 50 mM MgCl_2_, and 100 mM KCl). In addition, 20 mM l-alanine was added for the co-crystallisation of M2PYK/Ala crystals; 50 mM l-phenylalanine was added for M2PYK/Phe crystals; 100 mM l-serine, 1 mM oxalate, and 1 mM ATP were added for M2PYK/Ser crystals; 30 mM l-tryptophan was added for M2PYK/Trp crystals. The pH for obtaining optimal crystals was 7.2–7.8. Before data collection, crystals were dipped in a freezing solution consisting of reservoir solution supplemented with the same concentration of ligands as in the original solution, as well as up to 35% PEG 3350, which eliminated the appearance of ice rings. Diffraction data were collected at the Diamond synchrotron radiation facility in Oxfordshire, U.K. on beamline I03 (for the M2PYK/Ala structure), I04-1 (for the M2PYK/Phe and the M2PYK/Trp structures), and I24 (for the M2PYK/Ser structure). All datasets were obtained from a single crystal flash-frozen in liquid nitrogen. A more detailed description and discussion of the crystallisation trials is in the Supplementary Material.

### Structure determination

All four M2PYK structures were solved by molecular replacement using the program PHASER [40]. A monomer (Chain A) from the previously determined tetrameric structure of M2PYK (in R-state, PDB ID: 4B2D) served as the search model for the M2PYK/Ser structure. Chain A of a different M2PYK structure (in T-state, PDB ID: 4FXJ) was used as the search model for the M2PYK/Phe, M2PYK/Ala, and M2PYK/Trp structures. There were clear molecular replacement solutions for all structures. The resulting models were then subjected to 10 cycles of rigid body refinement using the program REFMAC [41]. Afterwards, manual changes were made to models to adjust side chain conformations and to add additional residues to the N terminus using COOT [42]. The models were then subjected to several rounds of translation libration screw refinement and restrained refinement, and ligand molecules were added where clear unbiased *F*_o_–*F*_c_ electron density was observed. Water molecules were added to the model using COOT, and after several rounds of restrained refinement, *R*/*R*_free_ values converged. Data collection and refinement statistics are summarised in Table 1.

**Table 1:** Data collection and refinement statistics

	M2PYK/Ala	M2PYK/Phe	M2PYK/Trp	M2PYK/Ser
Data collection				
Space group	*P* 2_1_ 2_1_ 2_1_	*P* 1 2_1_ 1	*P* 1 2_1_ 1	*P* 1
Cell dimensions				
*a*, *b*, *c* (Å)	160.72, 199.36, 243.23	97.37, 70.34, 168.64	96.88, 70.58, 168.50	93.35, 108.94, 124.34
*α*, *β*, *γ* (°)	90.00, 90.00, 90.00	90.00, 106.06, 90.00	90.00, 106.02, 90.00	89.72, 71.13, 66.94
Wavelength (Å)	0.9763	0.91741	0.91741	0.97625
Resolution^1^ (Å)	199.33–3.72 (3.82–3.72)	71.88–2.46 (2.524–2.46)	72.54–3.20 (3.283–3.20)	13.24–2.96 (3.04–2.96)
*R*_merge_^2^	0.125 (0.832)	0.077 (0.605)	0.196 (0.845)	0.074 (0.544)
*I*/*σI*	7.5 (1.8)	14.2 (2.5)	7.5 (1.4)	8.4 (1.8)
Completeness (%)	95.3 (96.3)	98.8 (99.6)	98.2 (99.4)	98.6 (98.0)
Multiplicity	4.2 (4.3)	5.8 (5.7)	2.9 (3.0)	2.7 (2.7)
Refinement				
*R*_work_/*R*_free_^3^	0.2196/0.2488	0.1966/0.2246	0.2284/0.2652	0.2360/0.2516
No. atoms	46 088	14 593	14 263	31 408
Protein	45 962	14 180	14 132	31 296
Ligand/ion	126	60	104	112
Water	0	353	27	0
*B*-factors				
Protein	138.2	63.8	59.0	93.4
Ligand/ion	118.0	68.0	61.0	94.0
Water	—	54.8	18.0	—
R.m.s. deviations				
Bond lengths (Å)	0.009	0.0085	0.013	0.0076
Bond angles (°)	1.299	1.272	1.496	1.163
Ramachandran plot (%)				
Favoured regions	95	98	96	95
Allowed regions	4	2	3	4
Disallowed regions	1	0	1	1
Conformation	T-state	T-state	T-state	R-state
PDB code	6GG3	6GG4	6GG5	6GG6

1 Values in parentheses are for the highest resolution shell.

2 *R*_merge_ = Σ*_hkl_*|*I−*〈*I*〉|/Σ*_hkl_I*.

3 *R*_work_ = Σ|*F*_obs_*−F*_calc_|/Σ|*F*_obs_|, where *F*_obs_ and *F*_calc_ are the observed and the calculated structure factors, respectively. *R*_free_ is calculated using 5% of total reflections randomly chosen and excluded from the refinement.

## Results

### Time-dependent dissociation of M2PYK tetramers

**Figure 2.**
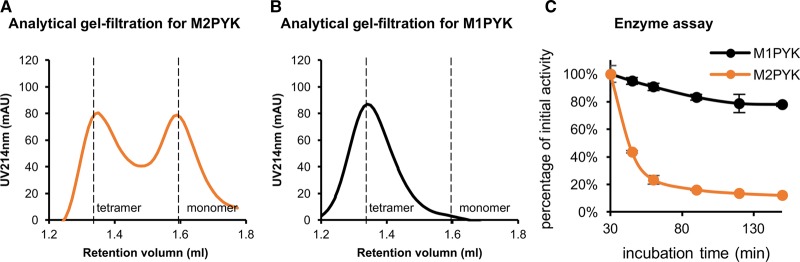
Analytical gel-filtration and enzyme assays suggest M2PYK equilibrates between tetramer and monomer, whereas M1PYK retains its tetrameric form. (**A** and **B**) Analytical gel-filtration assays for M2PYK and M1PYK (incubated for 12 h at room temperature) were carried out to determine the tetramer (left peaks): monomer (right peaks) ratio with a Superdex® 200 PC 3.2/30 gel-filtration column. (**C**) Gradual activity loss of M1PYK (black) and M2PYK (orange). Data represent the mean ± standard error of three experiments.

Gel filtration traces of M2PYK show two distinct peaks corresponding to the tetramer and the monomer (Figure 2A). The ratio of tetramer to monomer is concentration-dependent with higher concentrations favouring the tetrameric state. In this experiment, the time-dependent dissociation of M2PYK was measured by diluting a solution of 20 mg/ml M2PYK to 0.1 mg/ml (in PBS-CM, pH 7.4) and incubating for 0, 1, 2, 5.5, 8, and 12 h at room temperature (Supplementary Figure S2). The results indicate that M2PYK dissociated slowly from tetramer to monomer, reaching an equilibrium of 1 tetramer: 4 monomers after many hours. These gel filtration studies, carried out at different concentrations [13], also provide an estimate of a tetramer to monomer dissociation constant. Supplementary Figure S2 shows gel filtration traces for an M2PYK sample with a total protein concentration of 1.8 µM which after equilibration shows an equal (50:50 by mass) concentration of monomers (0.9 µM) and tetramers (0.23 µM). These equilibrium values are consistent with an apparent M2PYK tetramer–monomer dissociation constant of 0.9 µM. A similar experiment was carried out with the M1PYK isoform and showed no dissociation of the tetrameric form (Figure 2B). Gel filtration studies can only provide reliable signal at relatively high protein concentrations greater than ∼0.1 mg/ml. M1/M2PYK concentrations in normal and cancerous cells have been estimated at values between 0.005 and 2.6 mg/ml (Supplementary Figure S3) [38], which bracket the concentrations used for the gel filtration experiments.

Many papers have shown that the M2PYK tetramer has higher activity relative to its monomeric/dimeric forms [5,21,22,43,44]. Here, we show that loss of enzyme activity correlates with the time-dependent dissociation observed in the gel filtration experiments. In the enzyme assay (see Experimental Procedures) activities were measured using a stock solution of 20 mg/ml M2PYK which was diluted to a concentration of 0.002 mg/ml in PBS-CM (pH 7.4). Monitoring enzyme activity as a function of time showed a 50% loss of activity of M2PYK in 15 min (Figure 2C). The enzyme concentration used to measure the enzyme activity is 50 times more dilute than the solutions studied by gel filtration. An identical study with M1PYK showed only 17% loss of M1PYK activity over the 3 h period of the experiment consistent with M1PYK retaining its active tetrameric structure even at very low protein concentration. The time and concentration dependencies on the activity of M2PYK also explain apparent differences with previously published results on the effect of amino acids on M2PYK measured using end-point assays (see Supplementary Material).

### Amino acids have significant effects on the activity of M2PYK but not M1PYK

Here, we show the regulatory effects of physiological amino acids on M2PYK which were compared with those of M1PYK under the same conditions. PYKs were incubated with 2.5 mM amino acids including alanine, arginine, asparagine, aspartic acid, cysteine, glutamic acid, glutamine, glycine, histidine, isoleucine, leucine, lysine, methionine, phenylalanine, proline, serine, threonine, tryptophan, tyrosine and valine (all L-stereoisomers) or with no amino acid (as control). FBP was used as a positive control. The reaction was performed after pre-incubation in the presence of sub-saturating substrates in PBS at pH 7.4 at 37°C as described in Experimental Procedures.

**Figure 3.**
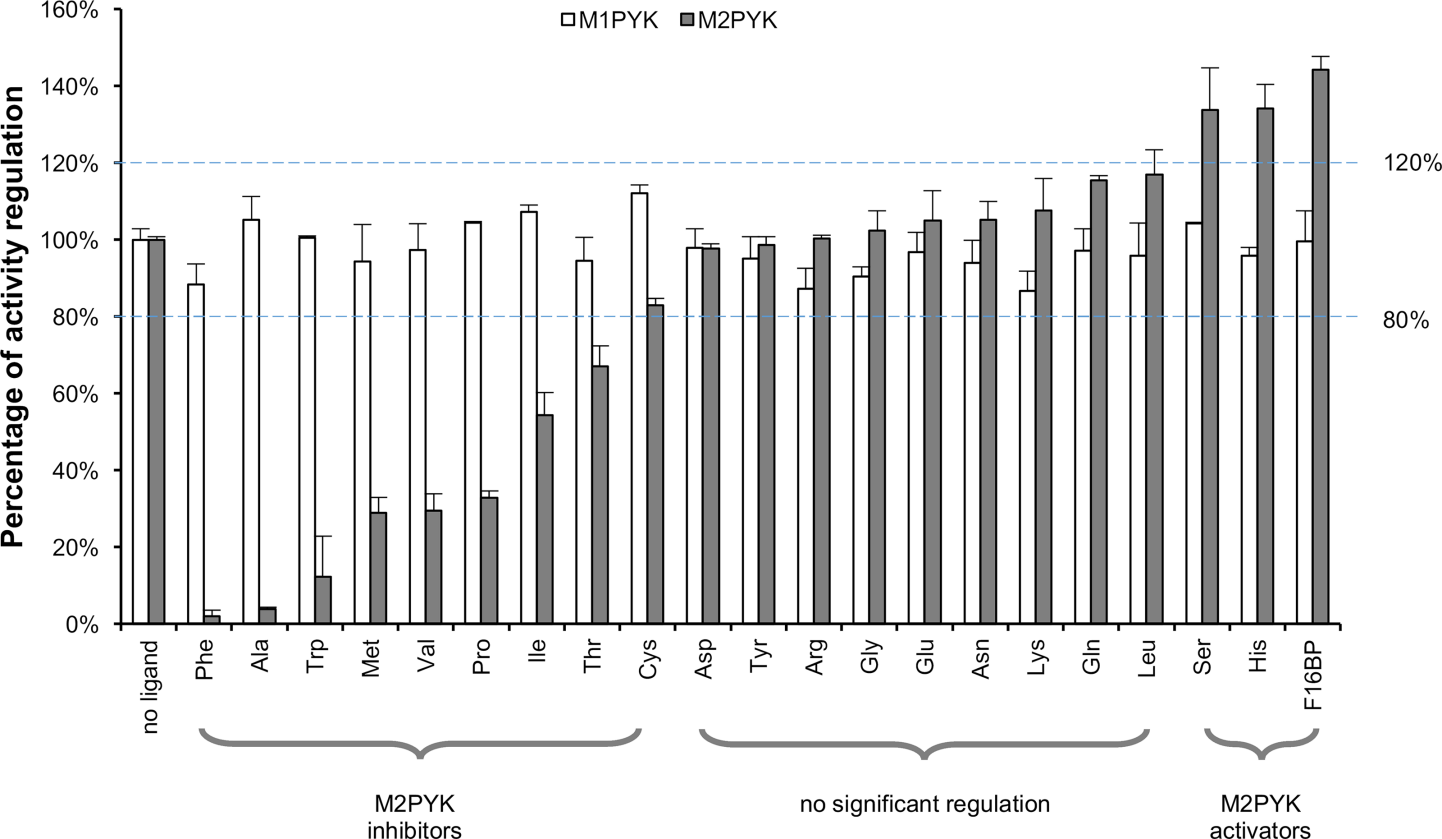
Effects of amino acids and FBP on the activity of M1PYK (□) and M2PYK (▪). The activities of M1/M2PYK in the absence of ligands were calculated as 100%, to which the activities tested in the presence of 2.5 mM ligands were normalised. Phenylalanine, alanine, tryptophan, methionine, valine, and proline were strong inhibitors for M2PYK (99–65%), whereas the inhibition by isoleucine, threonine, and cysteine was more modest (50–20%). Serine and histidine were identified as activators (higher than 120% activity) for M2PYK. The values within the dashed blue lines (100% ± 20%) may be regarded as showing no significant regulation. Data represent the mean ± standard error of three experiments. Sub-saturating concentrations of both substrates PEP (0.4 mM) and ADP (0.5 mM) were used.

Many amino acids showed strong effects on M2PYK activity (Figure 3). Phenylalanine and alanine were the most powerful inhibitors (nearly 100% inhibition) among all tested amino acids. Methionine, proline, tryptophan, and valine were also strong inhibitors (90–65% inhibition), whereas the inhibition by isoleucine, threonine, and cysteine was more modest (50–20% inhibition). Histidine and serine were identified as activators (∼30% activation) for M2PYK. As an extensively studied natural activator of M2PYK, FBP showed the strongest activation among all tested ligands (∼40% activation). In contrast, M1PYK (shown in white bars) was only slightly (10%) inhibited by phenylalanine, as well as by arginine, glycine and lysine, with all the other ligands, including FBP, having little or no regulatory effect. These results for M1PYK compare well with previous reports [13,29,33].

### Enzyme kinetics: amino acids are allosteric regulators of M2PYK

**Figure 4.**
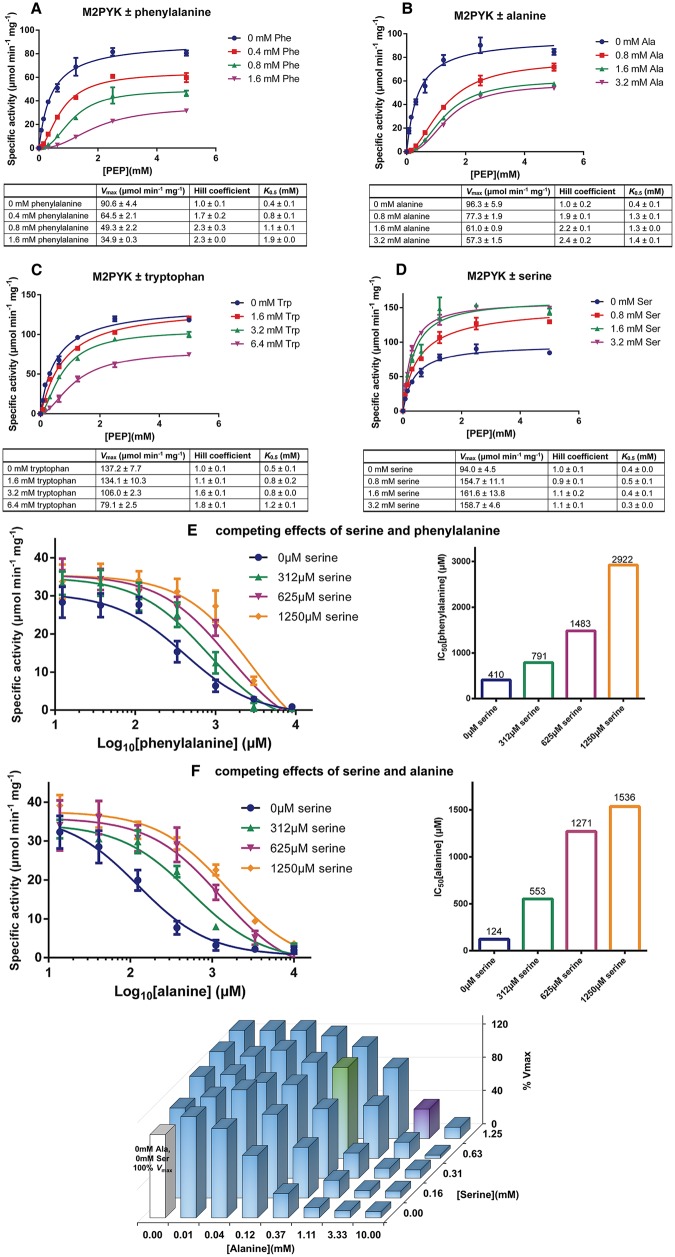
Effects of amino acids on M2PYK activity. (**A**–**D**) Kinetic profiles of M2PYK determined for PEP [with saturating ADP (2 mM)] in the presence or absence of different concentrations of phenylalanine, alanine, tryptophan, and serine. Lineweaver–Burk plots are shown in Supplementary Figure S4. (**E**) The competing effects of serine and phenylalanine on M2PYK activity. Sub-saturating concentrations of PEP (0.4 mM) and ADP (0.5 mM) were used for this enzyme assay. (**F**) The competing effects of serine and alanine on M2PYK activity. The 3D histogram on the right shows relative *in vitro* enzymatic activity of M2PYK in the presence of different concentrations of alanine/serine. The white bar is the M2PYK activity with no amino acid ligands, calculated as 100% activity. The green bar corresponds to M2PYK activity in the presence of alanine/serine at normal intracellular concentrations, while the purple bar corresponds to that of concentrations in cancer cells (Supplementary Figure S5). Sub-saturating concentrations of PEP (0.4 mM) and ADP (0.5 mM) were used for this enzyme assay. Data represent the mean ± standard error of three independent experiments.

To study the effects of the amino acids as regulators of M2PYK, we performed enzyme kinetic assays for PEP in the presence or absence of three inhibitors (phenylalanine, alanine, and tryptophan) and an activator (serine). The kinetic curves are shown in Figure 4 and Supplementary Figure S4. Rather than the sub-saturating concentrations of both substrates used for the modulator screening assay (Figure 3), a saturating concentration of ADP (2 mM) was used for the kinetics assay, giving an apparent *V*_max_ value of ∼100 µmol/min mg for M2PYK and a *K*_0.5_[PEP] of ∼0.4 mM. As shown in Figure 4A–C, addition of either of the three inhibitors significantly decreased *V*_max_ and increased *K*_0.5_[PEP], with a concomitant increase in co-operativity (the Hill coefficient increases from 1.0 to more than 2.0). The kinetic curves (Figure 4A–C) measured at the different inhibitor concentrations are consistent with phenylalanine, alanine and tryptophan each acting as allosteric uncompetitive inhibitors of M2PYK and show increasing concentrations of the PEP substrate failing to compete out the effect of these inhibitory amino acids. Although all three tested inhibitors show a similar inhibitory pattern on M2PYK, they have different IC_50_ values: alanine has the lowest IC_50_ of 124 µM compared with phenylalanine (410 µM) and tryptophan (445 µM). The inhibitor constants (*K_i_*) for alanine, phenylalanine, and tryptophan are 62, 205, and 222 µM, respectively. In contrast, serine activated M2PYK by increasing *V*_max_ (from 106 up to 170 µmol/min mg; Figure 4D). These results suggest that enzymatic activity of intracellular M2PYK may be affected by the relative concentrations of certain amino acids in the cytosol.

### Competitive effects of amino-acid activators and inhibitors at physiological concentrations

Different concentrations of binary mixtures of serine and alanine (or phenylalanine) were used to show their competing effects on enzyme activity (Figure 4E–F). At a concentration of 1250 µM, the activator serine increases the IC_50_ values of inhibitory amino acids alanine (from 124 to 1536 µM) and phenylalanine (from 410 to 2922 µM). The reported concentrations of amino acids in normal and cancer tissues are in the high micromolar to low millimolar range (Supplementary Figure S5) [45]. Phenylalanine at a cellular concentration of ∼0.2 mM reduces M2PYK activity to ∼65%. This value can be raised to nearly full activity in the presence of 0.6 mM serine (Figure 4E). With an approximate cellular concentration of alanine of 200–300 µM, M2PYK is only ∼10% active; however, in the presence of serine at an intracellular concentration of ∼0.6 mM, activity is restored to ∼65% (Figure 4F). An *in vitro* enzyme assay of M2PYK in the presence of different concentrations of both amino acids (Figure 4E–F) shows that in normal tissue, where the intracellular concentrations of alanine and serine are ∼0.5 and 0.6 mM, respectively, the activity of M2PYK is 109% (compared with unligated protein). Intracellular concentrations of amino acids can vary significantly within tumours [45] with alanine elevated up to 10-fold and serine by 2-fold compared with normal tissue. Figure 4F maps out the effect of these competing inhibitor and activator molecules on the enzyme activity of M2PYK in such an environment and shows that, depending on the relative (and absolute) concentrations of the amino acids, activity can be tuned from anywhere between 10 and 140%, providing a rapid mechanism for controlling the balance between cell growth and ATP production.

### ELISA and gel filtration assays show amino acids stabilise the tetramer form of M2PYK

**Figure 5.**
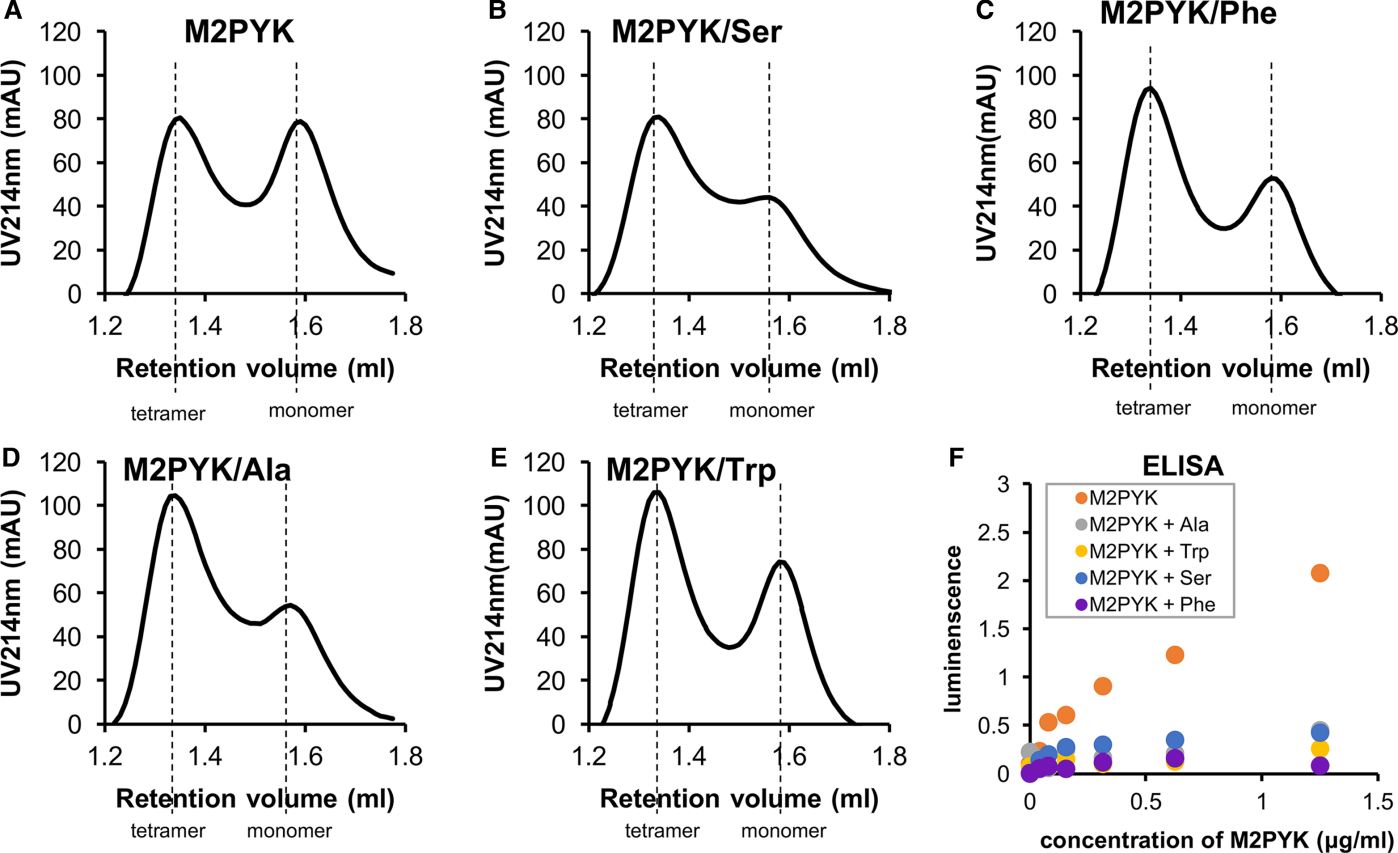
Amino acids and FBP minimise M2PYK dissociation. (**A**) Analytical gel chromatography for 0.1 mg/ml M2PYK incubated in PBS-CM (pH 7.4) for 12 h at room temperature (monitored at 214 nm). (**B**–**E**) Analytical gel chromatography for 0.1 mg/ml M2PYK incubated with 10 mM serine, phenylalanine, alanine, and tryptophan in PBS-CM (pH 7.4) for 12 h at room temperature (monitored at 214 nm). Dashed lines indicate the positions of elution of tetramers and monomers. (**F**) ELISA test using an anti-M2PYK antibody that binds to its C–C interface (shown in Figure 1). The values of luminescence reflect the amount of antibody that was bound to different concentrations of PYK in the presence of ligands (10 mM of each amino acid) at pH 7.4.

Analytical gel chromatography was used to investigate the effects of amino acids on the oligomerisation of M2PYK. Briefly, 0.4 ml samples of 0.1 mg/ml of M2PYK were incubated in the absence/presence of 10 mM alanine, phenylalanine, serine, or tryptophan in PBS-CM at pH 7.4 at room temperature for 12 h. The chromatograms (Figure 5) show that not only the activator serine, but also all the inhibitory amino acids stabilised tetramers over monomers. It is likely that alanine and tryptophan function by stabilising the T-state inactive tetramer in the same manner as that previously reported for phenylalanine [13], whereas serine induces the formation of an R-state tetramer. It has previously been shown that the M2PYK activator FBP stabilised the tetrameric form [13]. Dissociation studies of M2PYK using SEC-MALS [13] and ultracentrifugation [46] show the predominant forms in solution are tetramer and monomer. Size exclusion chromatography (Figure 5) shows no obvious peak that may correspond to M2PYK dimers. Earlier literature on M2PYK frequently refers to a tetramer–dimer equilibrium; however, all biophysical measurements in solution published to date suggest that tetramers and monomers are the predominant species.

To investigate the effect of ligand binding on monomer–tetramer equilibrium at lower protein concentrations, we developed an ELISA assay. A highly selective (M2PYK versus M1PYK) monoclonal antibody that only binds to the C–C interface of M2PYK was produced by immunising with an epitope specific for this region (Supplementary Figure S1). As the epitope is hidden in the tetrameric form of M2PYK, antibody binding on the C–C interface of M2PYK reflects the amount of monomer/dimer but not tetramer. The ELISA tests were performed using low concentrations of His_6_-M1/2PYK (0.0039–2.5 µg/ml) incubated with no ligand or 10 mM of each amino acid for 1 h in PBS-CM at pH 7.4 at room temperature. The incubated solutions were added to 96-well plates precoated with anti-His-tag antibodies. HRP-conjugated M2PYK antibodies were then incubated, followed by luminescence reading. The results (Figure 5F) showed that the reading for M2PYK in the absence of stabilising ligands was significantly higher than with alanine, phenylalanine, serine, and tryptophan. This indicates that, at low concentrations, M2PYK dissociates, thereby exposing the epitope on the C–C interface allowing the antibody to bind. In the presence of alanine, phenylalanine, serine, or tryptophan, M2PYK is predominantly tetrameric and the hidden epitope cannot be bound by the antibody.

### Crystal structures of amino acid complexes provide a mechanism of T- to R-state transition in M2PYK tetramers

The role of the natural effector FBP which binds 40 Å away from the active site (shown in Figure 1) of M2PYK has been extensively studied as a promoter of the R-state tetrameric form with concomitant activation [12,13,21,47]. Other reports have shown that M2PYK can transit into an inactive T-state tetramer [13,48]. Here, with the solution of wild-type M2PYK crystal structures co-crystallised with serine, alanine, phenylalanine, or tryptophan (Table 1, Figure 7 and Supplementary Figure S6), we demonstrate how different amino acids selectively stabilise M2PYK in tetrameric R or T states.

### R-state M2PYK/serine structure

**Figure 6.**
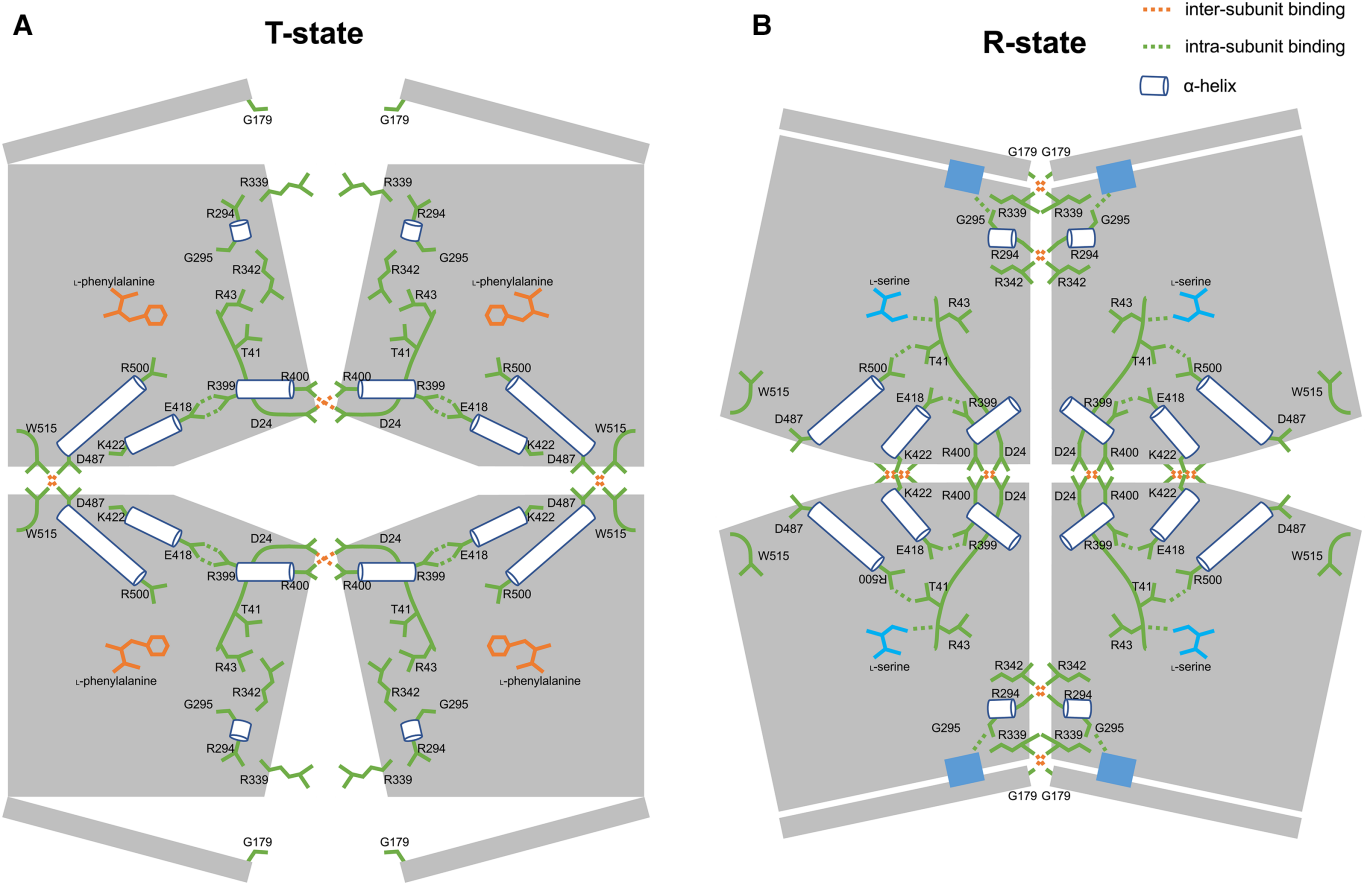
T-state and R-state M2PYK structure models. Each subunit of M2PYK is shown as an irregular pentagonal block. B-domains are represented by narrow rectangles. Amino-acid residues and loops are shown in green. Helices are shown as cylinders. Amino-acid inhibitor and activator are shown in orange and cyan, respectively. Important hydrogen-bonded and salt-bridge interactions are highlighted by dashed lines. Active sites are shown in blue rectangles.

Chaneton et al. obtained an R-state M2PYK structure in complex with serine [24] by soaking serine into an M2PYK/FBP crystal. FBP is a strong activator and locks M2PYK in the active R-state, and crystal soaking with serine leaves an open question as to whether the interactions made by serine are facilitated by the preformed FBP-bound R-state structure. Here, we co-crystallised serine with M2PYK in the absence of FBP and show that the serine interactions alone stabilise the R-state. An overlay of M2PYK/FBP (PDB ID 4FXF) with M2PYK/Ser gives an RMS fit (4 × 407 Cα atoms) for all four subunits of 0.93 Å (Table 2). This R-state conformation of M2PYK is very similar to the constitutively fully active M1PYK structure (RMS fit of 0.95 Å). The enhanced activity of the serine-bound structure can therefore be explained by the stabilisation of the R-state conformation (Figure 6) in which Arg342 from an adjacent protomer primes a glycine-rich active-site helix and locks it in a conformation allowing substrate binding [13]. The interactions made by serine in the amino-acid binding pocket help lock the R-state conformation which is stabilised by a well-defined arrangement of hydrogen bonds across the C–C and A–A interfaces (Figure 6).

**Table 2:** Comparisons of subunit rotation angles and tetramer fits among different M2PYK crystal structures 4FXJ: A published crystal structure with M2PYK-R489A and ligand phenylalanine. 4B2D: A published crystal structure with M2PYK/FBP/Ser. 4FXF: A published crystal structure with M2PYK/FBP. 3SRF: A published crystal structure with M1PYK.

	Ala	Phe	Trp	Ser	4FXJ	4B2D	4FXF	3SRF
Ala	* *	**3.23**	**3.66**	**9.1**	**3.56**	**9.25**	**9.19**	**9.29**
Phe	*1.45*		**0.47**	**11.28**	**0.38**	**11.23**	**11.12**	**11.76**
Trp	*1.58*	*0.26*	* *	**11.52**	**0.59**	**11.53**	**11.37**	**12.05**
Ser	*3.65*	*4.68*	*4.76*		**11.50**	**0.65**	**1.08**	**1.66**
4FXJ	*1.49*	*0.38*	*0.60*	*4.68*	* *	**11.40**	**11.25**	**11.99**
4B2D	*3.70*	*4.69*	*4.79*	*0.42*	*4.68*		**0.97**	**1.99**
4FXF	*3.33*	*4.23*	*4.33*	*0.93*	*4.24*	*0.93*	* *	**2.71**
3SRF	*3.88*	*5.00*	*5.10*	*0.95*	*5.01*	*1.06*	*1.55*	

Italics: RMS fit (Å) (tetramer–tetramer best fit, calculated with Cα atoms). Bold: subunit rotation angles (°) (calculated based on the tetramer–tetramer RMS best fit).

### Inhibitory alanine, phenylalanine, tryptophan T-state structures

**Figure 7.**
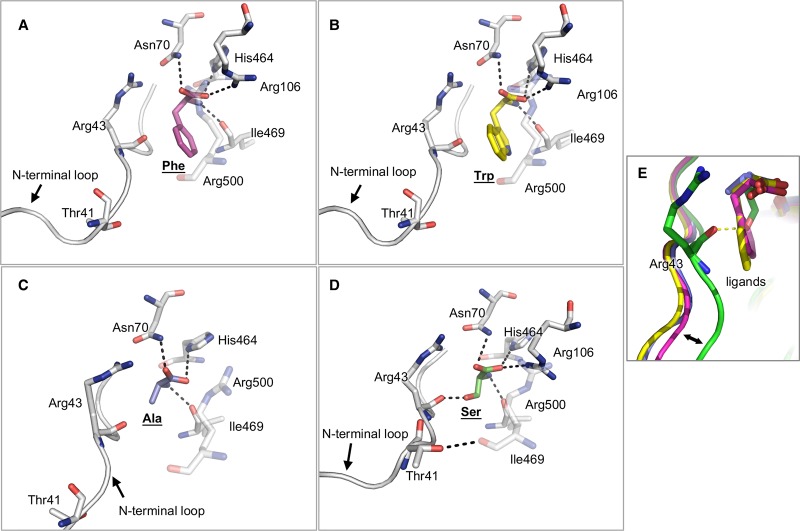
Effects of amino-acid binding on the conformation of the binding pockets. (**A**–**C**) Binding of inhibitory amino acids phenylalanine, tryptophan, and alanine, respectively. (**D**) Binding of the activator serine. (**E**) Comparison of the four binding conformations to highlight different structural effects of the amino acids on their binding pockets. The hydrophobic side chains of the inhibitors alanine, phenylalanine, and tryptophan push the N-terminal loop (residues 1–43) outwards, whereas the hydrophilic side chain of serine, an activator, stabilises the loop in an inward position by forming a hydrogen bond with Arg43.

Co-crystal structures of M2PYK with alanine, phenylalanine, or tryptophan show the amino acids all binding with similar poses in the amino-acid binding pocket (located between the A-domain and C-domain of each subunit). The carboxyl groups of the amino acids form strong hydrogen bonds to the side chains of Asn70 and Arg106, and the amino groups hydrogen-bond to the side chain of His464 and the main-chain oxygen on Ile469 (Figure 7). They adopt a binding pose that is very similar to that observed in the activating serine structure except that the hydrophobic side chains of the three inhibitory amino acids cause a widening of the binding pocket and make contact with the carbonyl oxygen atom from Arg43, effectively pushing away the N-terminal strand (Figure 7). In contrast, in the serine structure the side chain hydroxyl group makes a hydrogen bond with the Arg43 carbonyl side chain. A T-state structure of a mutated form of M2PYK (R489A) co-crystallised with phenylalanine has been reported previously (PDB ID: 4FXJ) [13]. The mutation (R489A) prevents binding of FBP in the effector site. Comparison with the co-crystal structures of wtM2PYK with phenylalanine, alanine, or tryptophan (Table 2) shows that the R-/T-state transition is regulated by amino acid binding and is not affected by the R489A mutation.

## Discussion

We have shown that the R-state M2PYK, stabilised by FBP and/or serine, adopts a very similar conformation to the constitutively fully active M1PYK (PDB ID: 3SRF, the RMS fits for R-state structures and M1PYK are within 1 Å; Table 2). The hydrogen-bonding patterns across the A–A and C–C interfaces are also completely conserved between these structures (Supplementary Table S2).

As described previously [13], the R-state conformation puts Arg342 from an adjacent protomer in a position to hydrogen-bond to backbone carbonyls of a short helical stretch (Arg294-Glu300) (Figure 6), thus shaping and priming the active site to accept the PEP substrate molecule. Though both FBP and serine appear to act as M2PYK ‘activators’, they are in fact simply stabilising the R-state conformation and also preventing dissociation of the tetramer into inactive monomer or dimer forms. The enzyme activities of M2PYK measured in the presence of the activators support this picture (Figure 4D), and both *K*_m_ and *k*_cat_ values never improve on those of M1PYK, which provides the ideal active conformation and seems to exist as a stable, constitutively fully active tetramer at most biologically relevant concentrations.

The X-ray structure of the M2PYK/Ser complex shows that the attractive pull of the N-terminal domain by the serine side chain shrinks the allosteric pocket and results in the formation of a hydrogen bond between Thr41 and Arg500 (Figure 7D). In this active-R-state conformation, both the C–C and A–A interfaces are zipped together by a total of 20 cross-interface hydrogen bonds (Figure 6 and Supplementary Table S2).

The T-state structure is held together by a rather different network of inter-protomer hydrogen bonds across the C–C and A–A interfaces (Figure 6). Binding of the inhibitory phenylalanine, tryptophan, or alanine stabilises the T-state structure, each protomer subunit is rotated by 11° relative to the R-state structures (Figure 6). The X-ray structures of the inhibitory amino-acid complexes show the N-terminal strand pushed by ∼3–4 Å compared with the R-state serine structure (Figure 7). The repulsive push from alanine, phenylalanine, or tryptophan leads to an interesting hydrogen-bonding rearrangement in which four Asp24-Arg400 salt bridges stabilising the C–C interface in the R-state switch partners to stabilise protomers across the A–A interface in the rotated T-state (Figure 6 and Supplementary Figure S7). The C–C interface in the T-state conformation is held by hydrogen bonds between Asp487 and Trp515, with the indole side chain anchored in a pocket further securing the T-state conformer (Figure 6 and Supplementary Figure S8).

**Figure 8.**
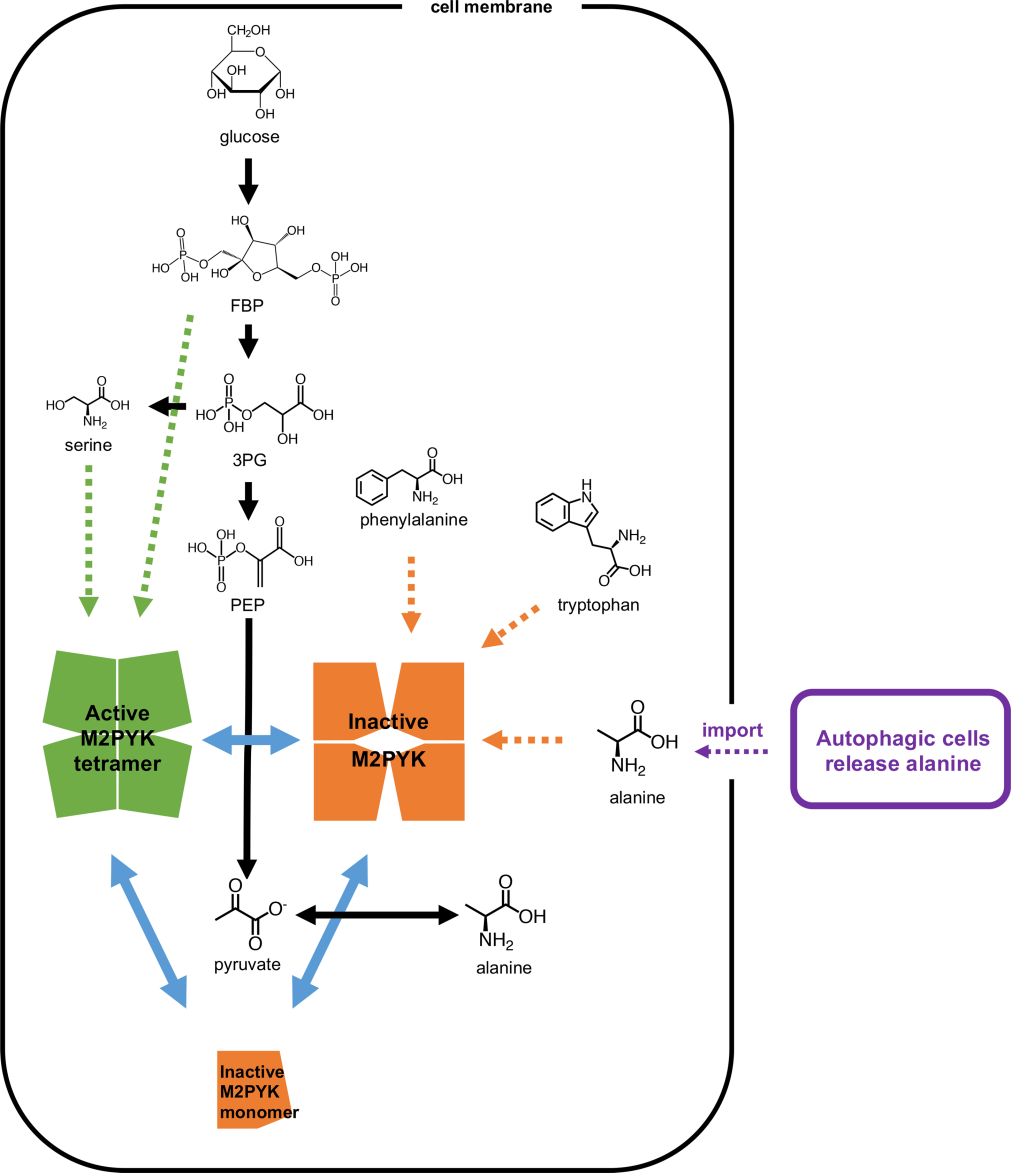
The regulation of M2PYK by metabolites. M2PYK is at the intersection of metabolic pathways (shown with black arrows). Blue solid arrows show the transformation of M2PYK among active tetramer (green), inactive tetramer (orange), and monomer (orange). Green dotted arrows show that FBP and serine stabilise M2PYK in the active tetrameric form, and thereby activate its enzymatic activity. In contrast, phenylalanine, tryptophan, valine, and alanine inhibit M2PYK enzymatic activity by stabilising it in an inactive tetrameric form (shown with orange dotted arrows). Autophagic alanine secretion from neighbouring cells [51] are shown in purple.

M2PYK is critical for regulating the synthesis and metabolism of serine. Knockdown and inactivation of M1 and M2 PYK have been shown to result in a build-up of metabolites of the glycolytic pathway, enabling synthesis of serine from its precursor 3-phosphoglycerate (Figure 8) [24]. In a complementary experiment, cells treated with a synthetic M2PYK activator became dependent on serine for continued cell proliferation [49]. Together with the knowledge that binding of serine activates M2PYK, these results suggested that there is a feedback mechanism regulated by serine concentration which provides a balance between glycolysis/ATP production (promoted by serine-bound active M2PYK) and cell proliferation (favoured by inactive M2PYK). This also fits with the result that M2PYK activated by FBP inhibits cell proliferation, while inhibition of M2PYK by phenylalanine favours cell proliferation [13].

Measured intracellular alanine concentrations from various cell types range from 0.4 mM to over 4 mM (Supplementary Figure S5), which are sufficient to inhibit M2PYK activity and stimulate (cancer) cell proliferation. The main biosynthetic route for alanine is via alanine transaminase (ALT) using pyruvate as a substrate [50], and elevated plasma levels of ALT are indeed found in cancer patients. Pancreatic ductal adenocarcinoma tumour cells rely on alanine, which feeds the tumour and which is provided in millimolar concentrations by autophagous pancreatic stellate cells [51]. Labelling studies further show that pyruvate generated from the transaminase feeds the tricarboxylic acid (TCA) cycle. These observations suggest that alanine serves a double purpose of decreasing glycolytic flux (allowing metabolite build-up) and also providing pyruvate for the TCA cycle.

The metabolic requirements of a cell committed to growth and division are very different from those of a quiescent or senescent cell. Once the fate of the cell is set, its metabolism must be reprogrammed accordingly. Homeostatic mechanisms that use feedback loops to maintain steady-state concentrations and activities need to be modified to accommodate the new environment: allostatic mechanisms facilitate those required changes in regulatory mechanisms. M2PYK therefore provides an excellent example of an allostatic regulator in which the opposing effects of activating (serine, histidine) and inhibiting (alanine, phenylalanine, tryptophan) amino acids provide a finely balanced feedback mechanism for tuning M2PYK activity over a wide range of absolute and relative concentrations. With high levels of serine (or FBP), M2PYK will be turned up and the TCA cycle will be fuelled by pyruvate from the last step in glycolysis [24]. With high levels of alanine (or phenylalanine or tryptophan), M2PYK is turned down and the TCA cycle may be fuelled by pyruvate produced by ALT [51] (Figure 8). Depending on the relative concentrations of serine (up to ∼1 mM) and alanine (up to ∼5 mM), M2PYK activity can be tuned to run from 5 to 140% of its velocity (Figure 4E). The important regulatory factor is the ratio of activating and inhibiting amino acids and not their absolute concentrations. Thus, M2PYK can act to regulate metabolism by sensing a range of concentrations and can use these inputs to control its enzyme activity by switching quickly (within seconds) between inactive T-state and active R-state conformations. At very low levels of amino-acid concentration (less than their *K_i_* values) and with enzyme concentration less than ∼2 µM, M2PYK will dissociate into enzymatically inactive monomers. Monomeric M2PYK has been shown to enter the nucleus and promote transactivation of HIF-1 target genes [19], many of which are metabolic enzymes, thus providing another regulatory mechanism for M2PYK.

The allostatic adjustments made by an organism to compensate for any adaptive change come at a cost that is defined as ‘allostatic load’. This (usually energetic) load is related to the stress imposed on the organism adjusting to its new state [52]. Here, we extend these ideas to encompass the allostatic load experienced by cells that are reprogrammed for proliferation (or senescence). For example, in cancer cells, biochemical pathways including metabolism are programmed for cell division and require a catabolic activity to match nutrient supply. Here, we show that M2PYK can sense the relative levels of different types of nutrient and process the various input signals to reduce the allostatic load on the cell. M2PYK is therefore able to guide the cell towards different fates: enhanced oxidative phosphorylation (by pyruvate production) or enhanced cell growth (by accumulation of other metabolites) or modified transcription patterns (by inactive monomers of M2PYK entering the nucleus [14]).

Cellular biochemical networks for metabolism, phagocytosis, and transcription are highly interconnected via various nodes. Regulation of the different cellular processes involves communication between the networks by perturbing molecular concentrations of their intermediates and by modulation of reaction rates of key enzymes. The structural and enzymatic results presented here provide a molecular description of the M2PYK metabolic sensor mechanism that can react to widely fluctuating nutrient concentrations in the cell and deliver appropriate allostatic outputs to these various biochemical processes.

## Abbreviations

ALT: alanine transaminase
ELISA: enzyme-linked immunosorbent assay
FBP: fructose 1,6-bisphosphate
HRP: horseradish peroxidase
LDH: lactate dehydrogenase
M1PYK: M1 isoenzyme of pyruvate kinase
M2PYK: M2 isoenzyme of pyruvate kinase
PBS-CM: Dulbecco's phosphate-buffered saline without calcium and magnesium
PDB: Protein Data Bank
PEP: phosphoenolpyruvate
PYK: pyruvate kinase
TCA: tricarboxylic acid
