# Supplementary material for: An allostatic mechanism for M2 pyruvate kinase as an amino-acid sensor
Source: Biochem J. 2018 May 31;475(10):1821–37. doi: 10.1042/BCJ20180171 (PMC5980995; doi:10.1042/BCJ20180171)
Supplement: Supplementary Tables and Figures [file BCJ-475-1821-s1.pdf]

SUPPLEMENTARY MATERIALS

Comparison among M2PYK structures co-crystallised with different amino acids

Wild-type M2PYK was co-crystallised with different amino acids in similar conditions. The concentration for PEG 3,350 was optimised in the range of 9% – 18%. The pH was optimised from 6.7 to 8.2. Near-solubility concentrations were used for each amino acid at first, which gave diffractable crystals except alanine. After the alanine concentration trial (from 10 – 100 mM), a relatively low concentration of 20 mM alanine was chosen. This is because alanine is a structural analogue of PEP, which might be able to bind into the active site at high concentration and interfere with the crystal growth.

The optimal condition for each crystal growth is listed in Table S1. The crystal of M2PYK with the activator serine was grown into a plate shape. Crystals grown with all the three inhibitors, alanine, phenylalanine, and tryptophan, were all in bar shapes. The crystal growth process and parameters (eg. unit cells, space groups, etc., shown in Table S1) of M2PYK/Phe and M2PYK/Trp are almost identical. However, although alanine inhibits M2PYK in an enzymatically similar way as phenylalanine or tryptophan, the crystal of M2PYK/Ala was rather different. First, M2PYK/Ala crystals favoured higher pH (pH 7.8 – 8.0). Second, molecules packed in different ways. For the M2PYK/Ala crystal structure, three asymmetric tetramers (i.e. twelve chains) were found in one unit cell (space group: $P\ 2_1\ 2_1\ 2_1$). On the contrary, only one tetramer (i.e. four chains) was found in one unit cell (space group: $P\ 1\ 2_1\ 1$) in M2PYK/Phe or M2PYK/Trp crystal structures. Third, in the R-state crystal structure co-crystallised with serine, the N-terminal region (residues 1-12) was too disordered to solve. In T-state structures with phenylalanine or tryptophan, the disordered region was even longer (residues 1-22). To our surprise, although the M2PYK/Ala structure is also in T-state,

the N-terminal loop was more stable, and thus was solved in the crystal structure (start from residue Pro5). As far as we know, this is the longest N-terminal loop solved in all published M2PYK structures up to now. We show the comparison of the N-terminal loops from these structures in different conformations (Figure S9).

As shown in Figure S9, in the R-state structure stabilised with serine (green), an ‘engaged region’ was formed with electrostatics between Gln16-Gln17 and Asp34-Ser37-Pro38. As a result, the Gln16-Gln17 region was stabilised and thus ordered. Binding with the inhibitors, the hydrophobic side chains of phenylalanine (purple) and tryptophan (yellow) push the N-terminal loop, thereby flip the Ile40 which blocks the engaged region. In consequence, the Gln16-Gln17 fragment gets disordered. This is further confirmed by the M2PYK/alanine structure (blue), in which the ‘engaged region’ is disrupted so that it adopts another conformation. This region is further stabilised by the salt-bridge formed with His6 and Glu282 on the adjacent tetramer. These facts provide probable interpretations of why (1) the N-terminal loop in the M2PYK/Ala structure is more ordered than other structures; (2) M2PYK/Ala unit cells packed in three tetramers; (3) M2PYK/Ala structure favoured high pH.

Comparison to a previously published study measured the effect of amino acids on M2PYK activity

A previously published study measured the effect of the 20 amino acids on M2PYK activity using a Kinase Glo assay (Chaneton *et al.* 2012). In this ‘end-point assay’ the M2PYK concentration was 4 nM and only serine and FBP showed activation; all other amino acids showed a slightly inhibitory effect. As we have shown from our gel filtration and ELISA experiments (Figure 2), the dissociation of M2PYK to monomers is strongly concentration and time dependent with a dissociation constant of ~0.9 µM (Figure S2) and tetramer dissociation $t_{1/2}$ of 15 min (Figure 2C). At equilibrium, with a concentration of 4 nM, less than 5% of M2PYK is calculated to exist as active tetramer. The changes

in M2PYK activity measured at very low enzyme concentrations with long incubation times are unlikely to reflect the activation or inhibition effects of amino acid binding under more physiological conditions: with cellular concentrations between 0.1 and 1 mg/ml (Supplementary Figure 3) M2PYK is calculated to exist mainly (50% to 90%) in tetrameric form. To partially overcome this problem, we used a continuous assay with a higher concentration of M2PYK (120 nM) prepared by rapidly diluting a concentrated (360 µM) M2PYK stock solution. The final diluted assay solution incubated for 10 minutes is calculated to have over 35% of M2PYK in its (active) tetrameric form.

Table S1. Optimal conditions for the co-crystallisation of M2PYK with each amino acid

	M2PYK/Ala	M2PYK/Phe	M2PYK/Trp	M2PYK/Ser
Crystallisation method	Hanging drop	Hanging drop	Hanging drop	Hanging drop
Temperature (K)	290.15	290.15	290.15	290.15
Drop size	3 µl	3 µl	3 µl	3 µl
Duration of crystal growth	7 days	42 days	42 days	65 days
M2PYK (mg/ml)	10	10	10	10
amino acid (mM)	20	50	30	100
PEG 3,350	11%	16%	15%	14%
sodium cacodylate (mM)	50	50	50	50
TEA (mM)	20	20	20	20
PO_4 (mM)	5	5	5	5
Na^+ (mM)	70	120	120	120
K^+ (mM)	50	50	50	50
Mg^{2+} (mM)	25	25	25	25
ATP (mM)	0	0	0	1
oxalate (mM)	0	0	0	1
pH	7.8	7.4	7.4	7.2

Table S2. Interface bonds of M1PYK and M2PYK.

**M2PYK/Phe A-Ainterface**		**M2PYK/Ser A-Ainterface**		**M1PYK A-Ainterface**		**M2PYK/Phe C-Cinterface**		**M2PYK/Ser C-Cinterface**		**M1PYK C-Cinterface**	
Chain A	Chain B	Chain A	Chain B	Chain A	Chain B	Chain A	Chain D	Chain A	Chain D	Chain A	Chain D
C31	R316	C31	R316	C31	R316	D487	W515				
D24	R400					D487	R516				
D347	R342					E484	R516				
D347	S346	D347	S346	D347	S346			D24	R400	D24	R400
D36	H274	D36	R278	D36	H274					M22	R400
E28	R319	E28	R319	E28	R319			E396	R400		
E304	R339							E418	R399		
E384	K305	E384	K305	E384	K305			K422	P403		
E397	L27	E397	L27	E397	L27					S402	K422
E397	F26	E397	F26	E397	F26					S405	K422
		G179	R339	G179	R339	M525	M525	M525	M525	M525	M525
		G295	R342	G295	R342	N523	V527	N523	V527	N523	V527
		G298	R339	G298	R339						
H274	D36	H274	D36	H274	D36						
I35	K305	I35	K305	I35	K305						
K305	E384	K305	E384	K305	E384						
K311	N350	K311	N350	K311	N350						
L27	E397	L27	E397	L27	E397						
Q329	R342	Q329	R342	Q329	R342						
Q393	Q393	Q393	Q393	Q393K							
R278	D36	R278	D36								
		R294	R342	R294	R342						

Interface bonds changed in different conformational states are highlighted in yellow. The Q393 in M2PYK that is replaced by K393 in M1PYK is highlighted in green.

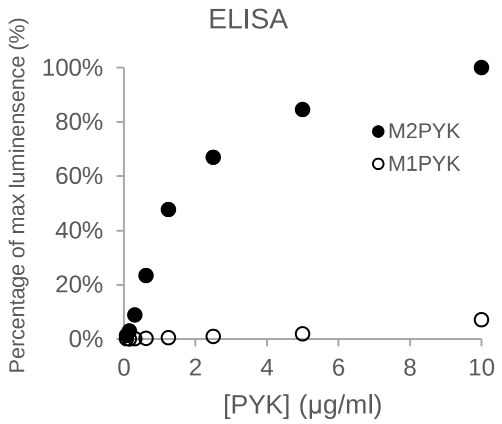

Figure S1. Determination of the specificities of anti-M2PYK antibody. M2PYK-specific epitope (amino-acid sequence: 'RRLAPITSDPT') was immunised in mice to generate specific monoclonal antibodies. Titrated M1PYK and M2PYK were coated onto a 96-well microtitre plate. Antibodies were incubated against each PYK.

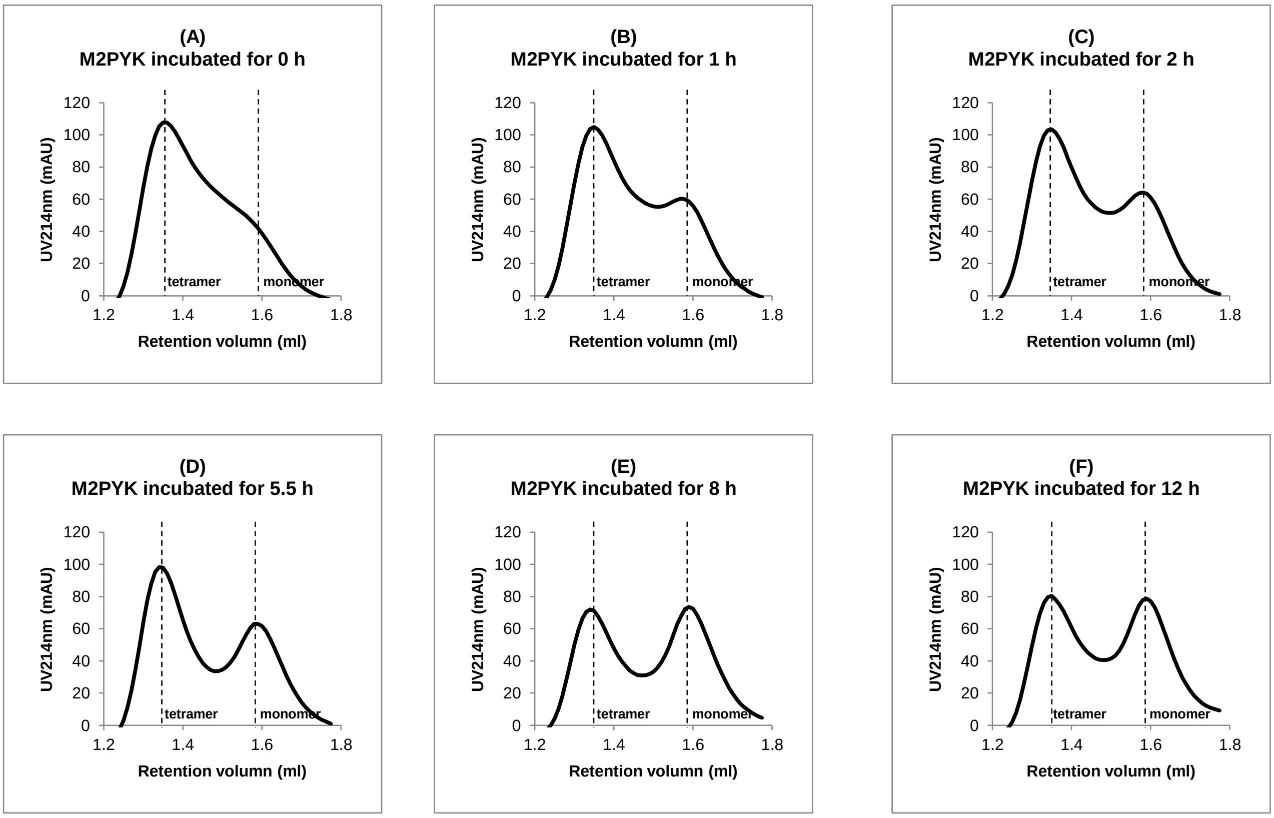

Figure S2. Time-dependent dissociation of M2PYK. **(A-F)** Analytical gel chromatography for 0.1 mg/ml M2PYK incubated in PBS-CM (pH 7.4) for 0 h, 1 h, 2 h, 5.5 h, 8 h, and 12 h at room temperature (monitored at 214 nm). The proportion of the tetramer peak decreased with time whereas the amount of monomer increased. The tetramer : monomer ratio reaches equilibrium after 8 h.

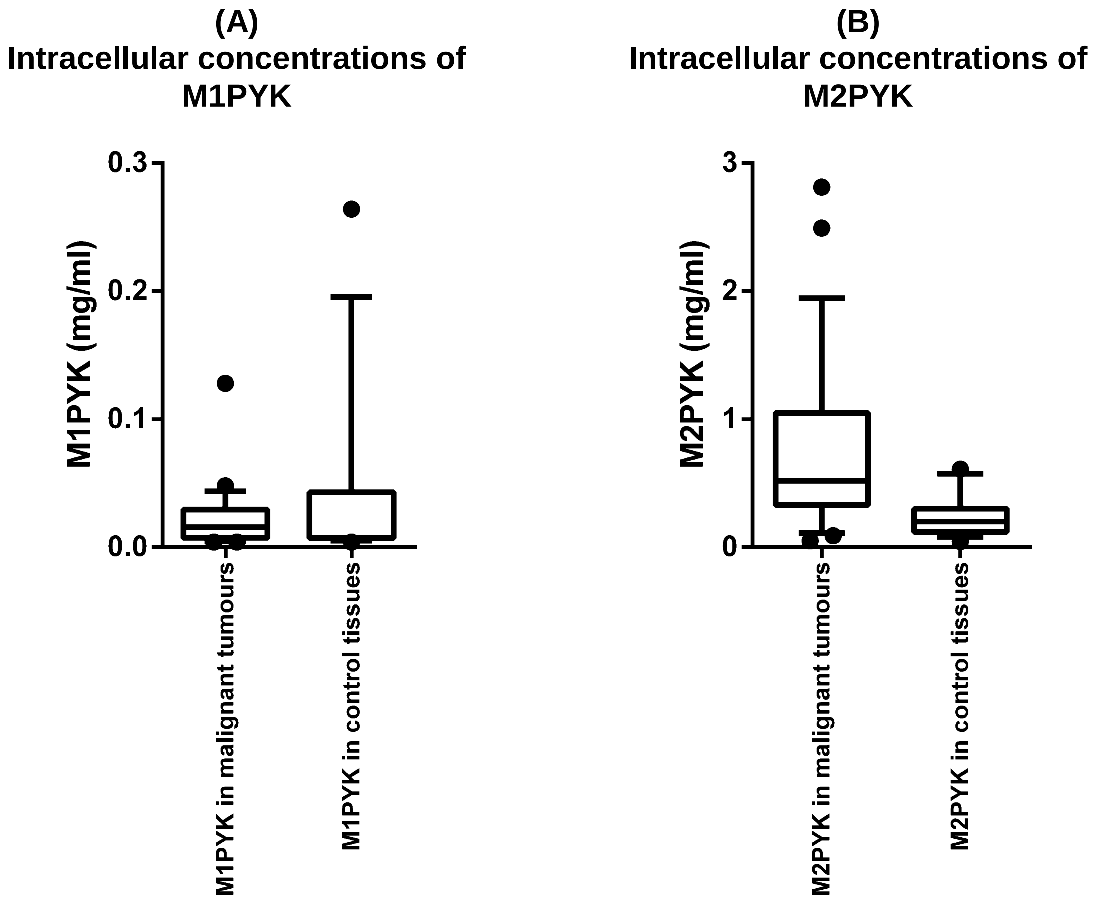

Figure S3. Intracellular concentrations of **(A)** M1PYK and **(B)** M2PYK. The data are calculated from Bluemlein *et al.* (2011). Non-cancer tissues (i.e. kidney, bladder, liver, colon, lung, and thyroid tissues) are used as the control tissues. The boxes extend from the 25th to 75th percentiles of each group. The whiskers are drawn down to the 10th percentile and up to the 90th. Points below and above the whiskers are drawn as individual dots. Horizontal lines inside each box represent the median numbers of each group. The median line of the group of 'M1PYK in control tissues' almost overlaps with the bottom line of this box.

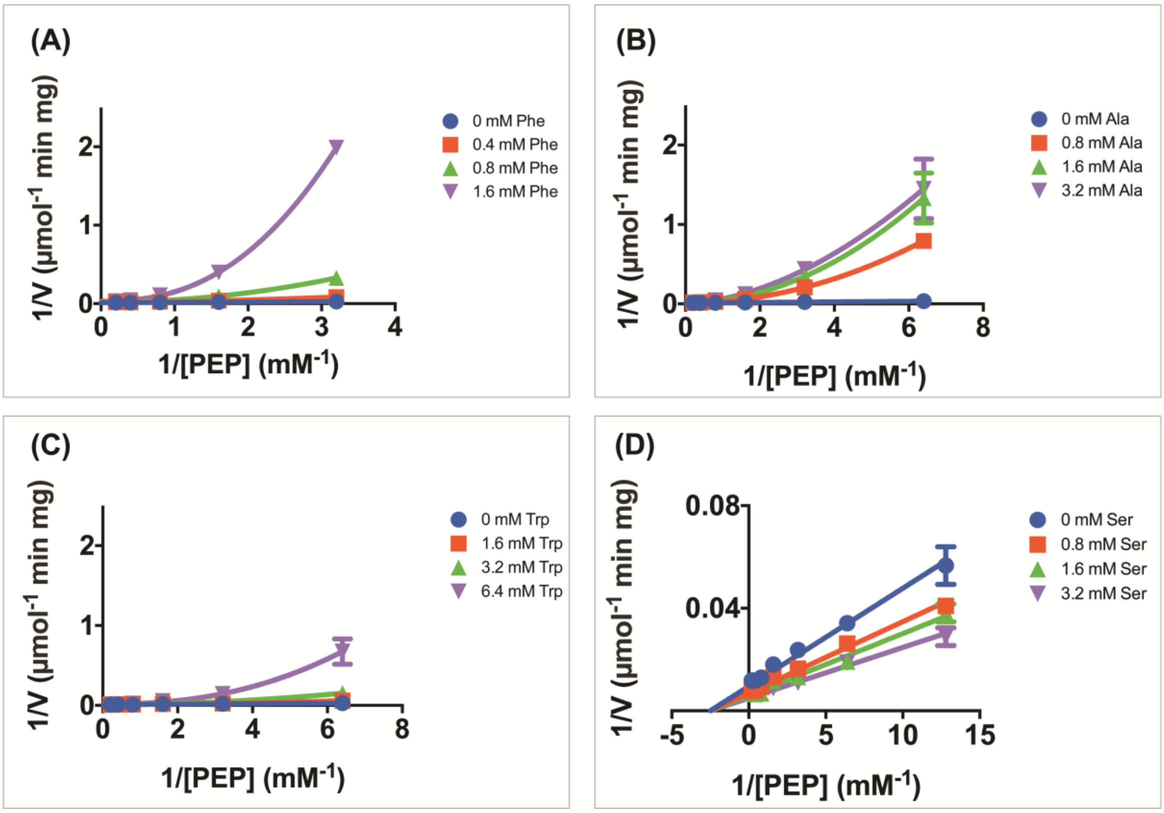

Figure S4. The Lineweaver-Burk plots of the kinetic profiles of M2PYK determined for PEP [with saturating ADP (2 mM)] in the presence or absence of different concentrations of **(A)** phenylalanine, **(B)** alanine, **(C)** tryptophan, and **(D)** serine. The non-linear Lineweaver-Burk plot suggests that the M2PYK-PEP catalysis is cooperative, and that the inhibitors enhanced its cooperativity. In other words, the inhibitors' allosteric effects and the PEP's allosteric effect to M2PYK were opposite.

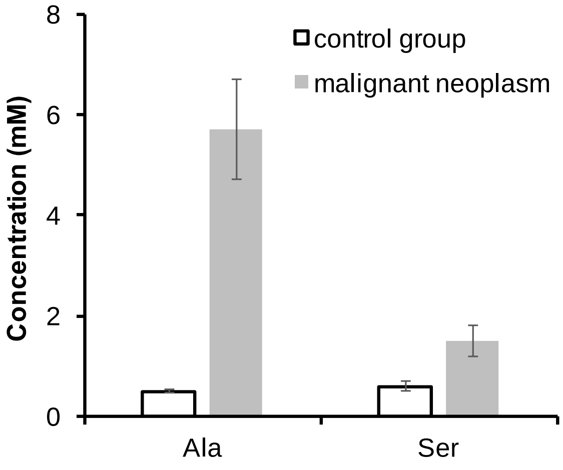

Figure S5. Intracellular concentrations of free amino acids in normal and tumour tissues. The concentration ranges of detected amino acids are from Brodzki *et al.* (2005).

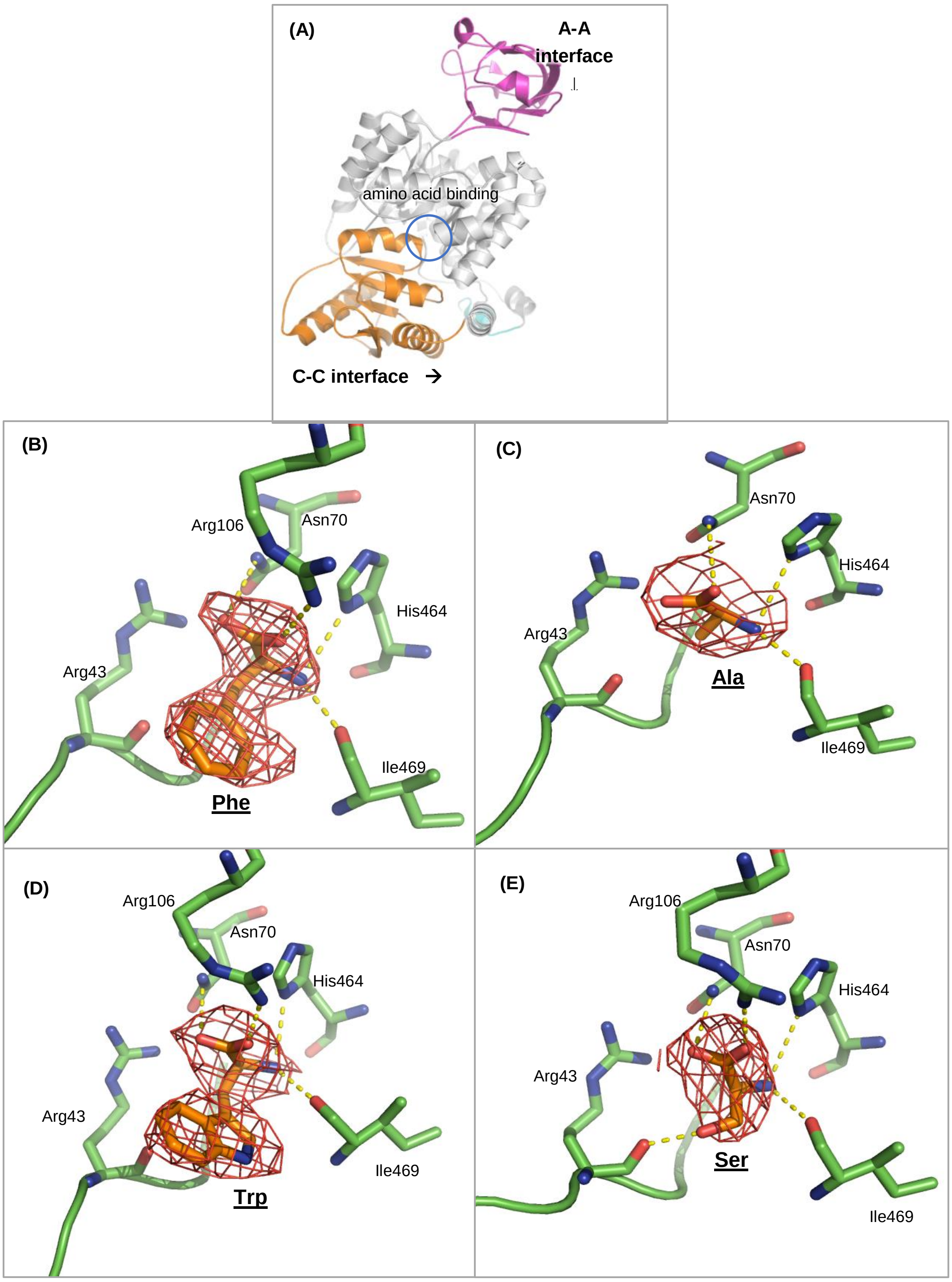

Figure S6. The bindings of amino acids on M2PYK. **(A)** Architecture of M2PYK monomer. N-terminus (cyan = residues 1–24); domain-A (grey = residues 25–116 and 220–402); domain-B (purple = residues 117–219); and domain-C (orange = residues 403–531). Location of the amino acid binding site is highlighted. **(B–E)** Structures of the amino acid binding site bound with Phe, Ala, Trp, and Ser, respectively. Hydrogen bonds and ion strength are shown in yellow dashed lines. The electron density of amino acid ligand in panel B, C, D, and E (coloured in orange) is from F_o−F_c maps contoured at the 3.0 σ, 1.8 σ, 2.4 σ, and 2.0 σ level, respectively.

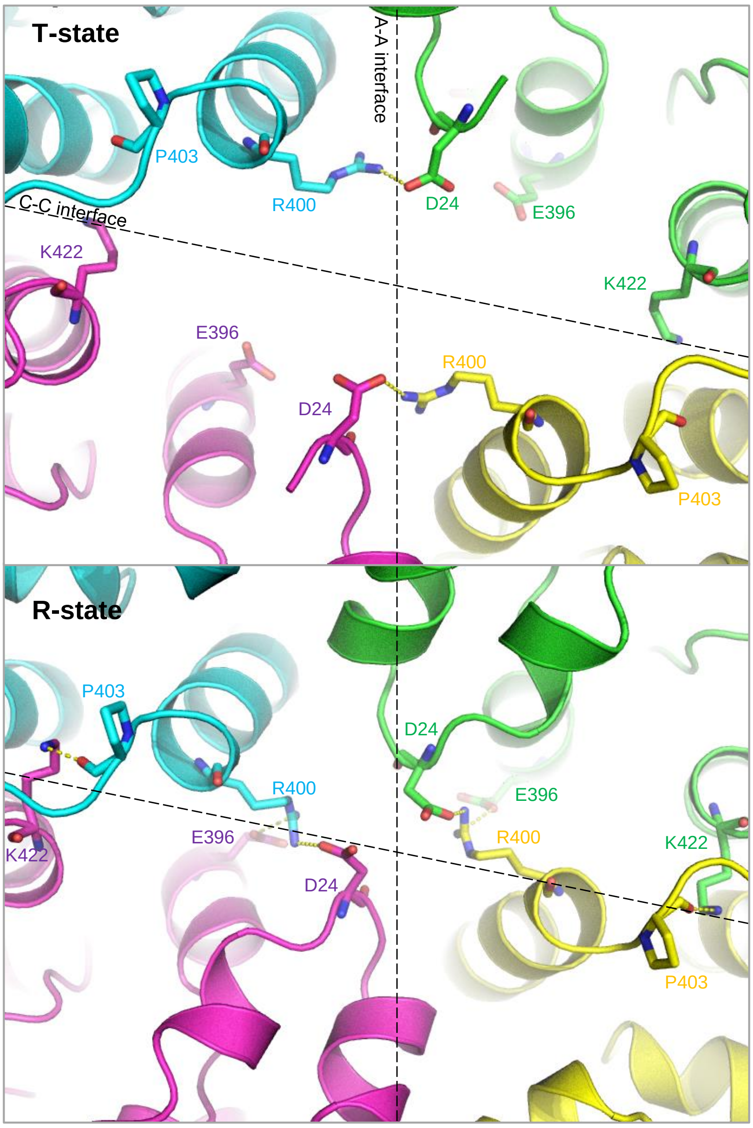

Figure S7. Interface bonds in the tetramer centre of M2PYK. The four subunits of the M2PYK tetramer are shown in different colours.

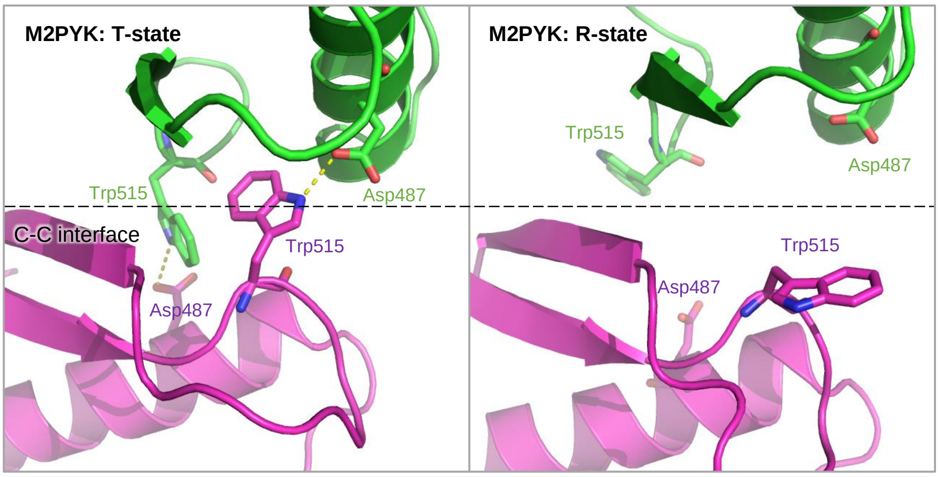

Figure S8. Interface bonds between Asp487 and Trp515 on the C-C interface of T-state M2PYK.

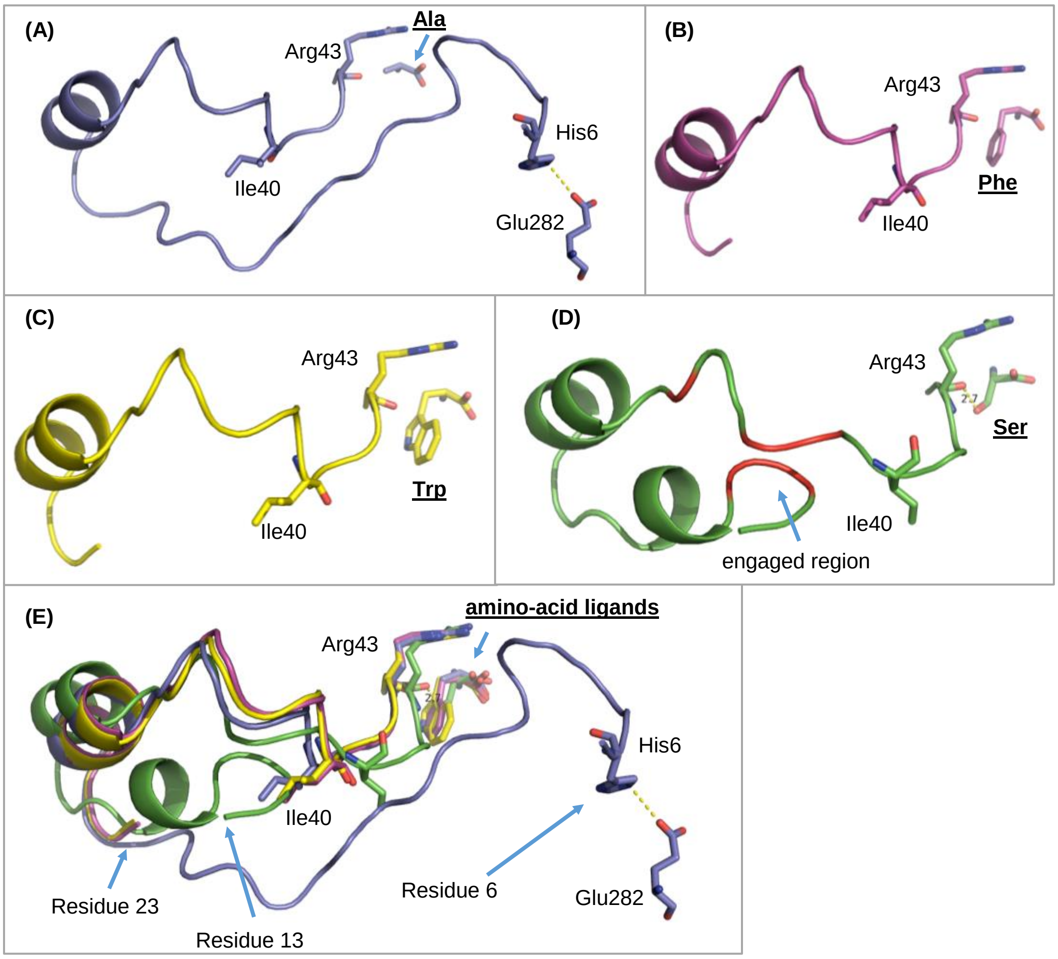

Figure S9. **(A-D)** N-terminal conformations of M2PYK/Ala structure (blue), M2PYK/Phe structure (purple), M2PYK/Trp structure (yellow), and M2PYK/Ser structure (green). Particularly, in panel **(D)**, the 'engaged region' formed with electrostatics between Gln16-Gln17 and Asp34-Ser37-Pro38 are highlighted in red. **(E)** Aligned N-terminal conformations. N-terminal regions are too disordered to be modelled. The 'solvable' ends are indicated in the figure (Residue 6, 23, 23, and 13 for alanine, phenylalanine, tryptophan, and serine, respectively).

Reference

Bluemlein, K., Gluckmann, M., Gruning, N.M., Feichtinger, R., Kruger, A., Wamelink, M., et al. (2012) Pyruvate kinase is a dosage-dependent regulator of cellular amino acid homeostasis. *Oncotarget* **3**, 1356-1369 https://doi.org/10.18632/oncotarget.730

Brodzki, A., Tatara, M.R., Pasternak, K., Rozanska, D. and Szponder, T. (2005) Free amino acids in skin neoplastic tissues and serum in dogs. *Bull. Vet. Inst. Pulawy* **49**, 231-235

Chaneton, B., Hillmann, P., Zheng, L., Martin, A.C., Maddocks, O.D., Chokkathukalam, A., et al. (2012) Serine is a natural ligand and allosteric activator of pyruvate kinase M2. *Nature* **491**, 458-462 https://doi.org/10.1038/nature11540
